# Hydrogen Peroxide Preconditioning Promotes Protective Effects of Umbilical Cord Vein Mesenchymal Stem Cells in Experimental Pulmonary Fibrosis

**DOI:** 10.15171/apb.2020.009

**Published:** 2019-12-11

**Authors:** Tayebeh Mahmoudi, Kamal Abdolmohammadi, Hamed Bashiri, Mehdi Mohammadi, Mohammad Jafar Rezaie, Fardin Fathi, Shohreh Fakhari, Mohammad Ali Rezaee, Ali Jalili, Mohammad Reza Rahmani, Lobat Tayebi

**Affiliations:** ^1^Cellular and Molecular Research Center, Research Institute for Health Development, Kurdistan University of Medical Sciences, Sanandaj, Iran.; ^2^Department of Immunology, Faculty of Medicine, Kurdistan University of Medical Sciences, Sanandaj, Iran.; ^3^Department of Immunology, School of Medicine, Tehran University of Medical Sciences, Tehran, Iran.; ^4^Department of Medical Laboratory Sciences, Faculty of Paramedical, Kurdistan University of Medical Sciences, Sanandaj, Iran.; ^5^Department of Immunology, School of Public Health, Tehran University of Medical Sciences, Tehran, Iran.; ^6^Zoonoses Research Center, Research Institute for Health Development, Kurdistan University of Medical Sciences, Sanandaj, Iran.; ^7^Cancer and Immunology Research Center, Research Institute for Health Development, Kurdistan University of Medical Sciences, Sanandaj, Iran.; ^8^Marquette University School of Dentistry, Milwaukee, WI, 53233, USA.

**Keywords:** Pulmonary fibrosis (PF), Bleomycin (BLM), Mesenchymal stem cells (MSCs), Myeloperoxidase (MPO), Hydrogen peroxide (H_2_O_2_)

## Abstract

***Purpose:*** Idiopathic pulmonary fibrosis (IPF) is a progressive lung disorder with few available treatments. Mesenchymal stem cell therapy (MSCT), an innovative approach, has high therapeutic potential when used to treat IPF. According to recent data, preconditioning of MSCs can improve their therapeutic effects. Our research focuses on investigating the anti-inflammatory and antifibrotic effects of H_2_ O_2_ -preconditioned MSCs (p-MSCs) on mice with bleomycin-induced pulmonary fibrosis (PF).

***Methods:*** Eight-week-old male C57BL/6 mice were induced with PF by intratracheal (IT) instillation of bleomycin (4 U/kg). Human umbilical cord vein-derived MSCs (hUCV-MSCs) were isolated and exposed to a sub-lethal concentration (15 μM for 24 h) of H_2_ O_2_ *in vitro*. One week following the injection of bleomycin, 2×10^5^ MSCs or p-MSCs were injected (IT) into the experimental PF. The survival rate and weight of mice were recorded, and 14 days after MSCs injection, all mice were sacrificed. Lung tissue was removed from these mice to examine the myeloperoxidase (MPO) activity, histopathological changes (hematoxylin-eosin and Masson’s trichrome) and expression of transforming growth factor beta 1 (TGF-β1) and alpha-smooth muscle actin (α-SMA) through immunohistochemistry (IHC) staining.

***Results:*** Compared to the PF+MSC group, p-MSCs transplantation results in significantly decreased connective tissue (*P*<0.05) and collagen deposition. Additionally, it is determined that lung tissue in the PF+pMSC group has increased alveolar space (*P*<0.05) and diminished expression of TGF-β1 and α-SMA.

***Conclusion:*** The results demonstrate that MSCT using p-MSCs decreases inflammatory and fibrotic factors in bleomycin-induced PF, while also able to increase the therapeutic potency of MSCT in IPF

## Introduction

Idiopathic pulmonary fibrosis (IPF) is a progressive inflammatory lung disorder with a not well-known etiology. IPF is identified by high deposition of extracellular matrix (ECM) proteins—mainly collagen—in lung tissue.^1^ Despite its obscure etiology, cellular damage and oxidative stress can activate inflammatory processes and recruit immune cells into damaged lung tissue.^2-4^ These uninterrupted inflammatory processes result in provoking lung tissue and unintentional expression of pro-inflammatory cytokines, such as TNF-α, IL-1β, and IL-8.^3^ Along with reactive oxygen species,^5^ agents that facilitate apoptosis by inducing the activation of caspases—specifically caspase-3—have been produced in lung tissue to impede reconstituting inflamed fibrosis lung, therefore causing gradual loss of the lung tissue’s proper function.^6^ Furthermore, pro-fibrotic cytokines, such as transforming growth factor beta 1 (TGF-β1) and alpha-smooth muscle actin (α-SMA), are overexpressed by differentiated myofibroblasts during fibrosis process in the lung of patients with PF and in animal models of experimentally-induced fibrosis.^7-9^ Bleomycin (BLM) is a chemotherapeutic medication, which can cause PF via regulation of TGF-β1. Therefore, BLM has been used to induce IPF in mice in many interventional studies.^7-9^

Despite accomplished extensive studies completed to find a decisive treatment of IPF, no effective therapy has been found to cure pulmonary fibrosis (PF) to date. However, IPF is one of the main life-threatening diseases, as expected survival of these patients after initial diagnosis is approximately 3 years.^10,11^ Nowadays, cell therapy using mesenchymal stem cells (MSCs)—with regards to the immunomodulatory and regenerative effects of these cells—is a novel or promising opportunity for the treatment of diverse inflammatory, autoimmune and tissue damage diseases, specifically lung diseases.^12-17^ MSCs are recognized by their spindle‐shaped morphology and expression of mesenchymal markers (such as CD44, CD73, CD90, and CD105) but no hematopoietic markers (CD14, CD34, and CD45). These cells have self-renewal capacity and differentiation potential to various cellular lineages, such as adipocytes, osteoblasts, and chondrocytes.^18-21^ Based on several studies, MSCs reduce tissue injury through the production of mediators with anti-apoptotic, anti-inflammatory and anti-fibrotic features.^12,15,16^ MSCs can be obtained from fetal tissues, such as umbilical cord blood and placenta, or adult tissues, like bone marrow and adipose tissue.^22^ Because they have a longer telomere, fetal tissue-isolated MSCs have higher proliferation capacity than that of adult tissue-MSCs.^5^ The umbilical cord, as a rich source of MSCs, is readily available and contains more MSCs than other tissues.^23,24^

According to previous studies, anything that increases the migration ability and survival of MSCs results in a more effective treatment with stem cell therapy. Vital properties of transplanted stem cells—such as survival, migration, and tissue repair capacity—are influenced dramatically by *in vitro* preconditioning of MSCs. Widespread studies have been accomplished regarding *in vitro* preconditioning on MSCs, but there is little information provided that investigate the effects of preconditioning of these cells in experimental animal models.^14,25-30^ In the present study, the anti-inflammatory and anti-fibrotic effects of H_2_O_2_ preconditioned hUCV-MSCs were evaluated in a bleomycin-induced PF mice model.

## Materials and Methods

### Animals

Twenty-eight male mice of the C57BL/6 strain were purchased from Pasture Institute of Iran (Tehran, Iran) and kept in an animal house under standardized conditions. In the time of experiments, mice were 6-8 weeks-old with average weight 20-30 g. The mice were transported to the laboratory for acclimation to the environment 72 h prior to the start of the experiment.

### Induction of PF and grouping

Experimental PF was induced by bleomycin under anesthesia, as previously mentioned.^8,31^ The mice were classified into four groups (n = 7). One group received sterile PBS intratracheal (IT) injection, which was considered as a control group (Ctrl). PF group was injected with bleomycin (4 U/kg, suspended in 50 µL sterile PBS, IT). The other treatment groups of mice include the PF+MSC group, which received 2×10^5^ hUCV-MSCs by intratracheal injection 1 week after bleomycin injection, while the PF+pMSC group received 2×10^5^ preconditioned hUCV-MSCs intratracheally.

### Isolation, culture, and expansion of hUCV-MSCs

Before participating in the study, informed consent was completed by pregnant women. Umbilical cords were collected from cesarean operations within the healthy term (38-40 weeks) and held in Hanks balanced salt solution buffer containing 300 μg/mL streptomycin, 300 U/mL penicillin, and amphotericin B (1%) (Invitrogen Gibco); then UCs were transferred to the laboratory. The UC vein (UCV) was washed twice with Hanks balanced salt solution and loaded with collagenase IV (0.1%) (Invitrogen Gibco), then the ends of UCV were clamped and incubated (37°C, 5% CO_2_, 20 min). The segregated cells were rinsed with Dulbecco’s Modified Eagle’s media with low glucose (DMEM-LG) (Invitrogen Gibco) and centrifuged at 1500 RPM for 15 min. The cell pellets were suspended and cultured in DMEM-LG containing 15% fetal bovine serum (FBS) (Gibco, USA), 100 U/mL of penicillin, and 100 μg/mL of streptomycin, then incubated (37°C and 5% CO_2_). Initial media was replaced after 48 hours and non-adherent cells were omitted. Thereafter, this was performed every 48 or 72 hours. After achieving 80-90% confluence, MSCs were incubated with trypsin 0.05% (Sigma, USA) and 0.02% EDTA for new passage and were cultured until passage 4.

### Characterization of hUCV-MSCs by flow cytometry analysis

MSCs at the fourth passage were trypsinized, washed using phosphate buffersaline (PBS) and resuspended in PBScontainingFBS (1%). A 100 μL aliquot of suspended MSCs was incubated for 45 minutes at 4°Cwith one of the following anti-human monoclonal antibodies (mAb): phycoerythrin (PE)-conjugated CD105, CD34, and CD73, or fluorescein isothiocyanate (FITC)-conjugated CD45 (BioLegend, USA). In addition, the mouse isotypic antibodies, including PE-IgG1j and FITC-IgG1j (BioLegend, USA), were utilized as control. After labeling of cells, they were evaluated using BD FACS Calibur™ flow cytometer (BD, USA) and analyzed using Flow Jo 7.6 Software.

### Characterization of hUCV-MSCs by differentiation assay

Human UCV-MSCs differentiation abilities into osteocyte and adipocyte lineages were examined at second passage.

### Osteogenic differentiation

MSCs were cultured at a density of 1×10^4^ cells/well in 24-well plates (SPL, Korea) and incubated at 37°C. After 24 hours, osteogenic differentiation media containing glycerol phosphate (10 mM), dexamethasone (100 mM) and ascorbic acid‐2 phosphate (5 g/mL) were added to cells every 72 hours for 3 weeks. After fixing the cells with 4% paraformaldehyde, Alizarin Red S staining utilized for detecting the mineralization in these cells.

### Adipogenic differentiation

1.5 × 10^4^ hUCV-MSCs/well were seeded in 24-well plates (SPL, Korea) and incubated at 37°Cfor 24 hours. Adipogenic differentiation media containing indomethacin (100 mM), 3‐isobutyl‐methylxanthine (0.5 mM), dexamethasone (250 mM) and insulin (5 mM) were added to cells every 3 days and incubated at 37°C for 2 weeks. After fixing the cells with 4% paraformaldehyde, Oil Red O staining utilized for determination of adipose vacuoles in these cells.

### Preconditioning of hUCV-MSCs with H_2_O_2_

MSCs were preconditioned with 15 μmol H_2_O_2_ for 24 hours, as previously reported.^27^ This non-toxic concentration of hydrogen peroxide (15 μmol) induces a protective influence on MSCs against the lethal dose of H_2_O_2_ (300 μmol).

### Transplantation of hUCV-MSCs to experimental models (treatment procedure)

**Figure 1:**
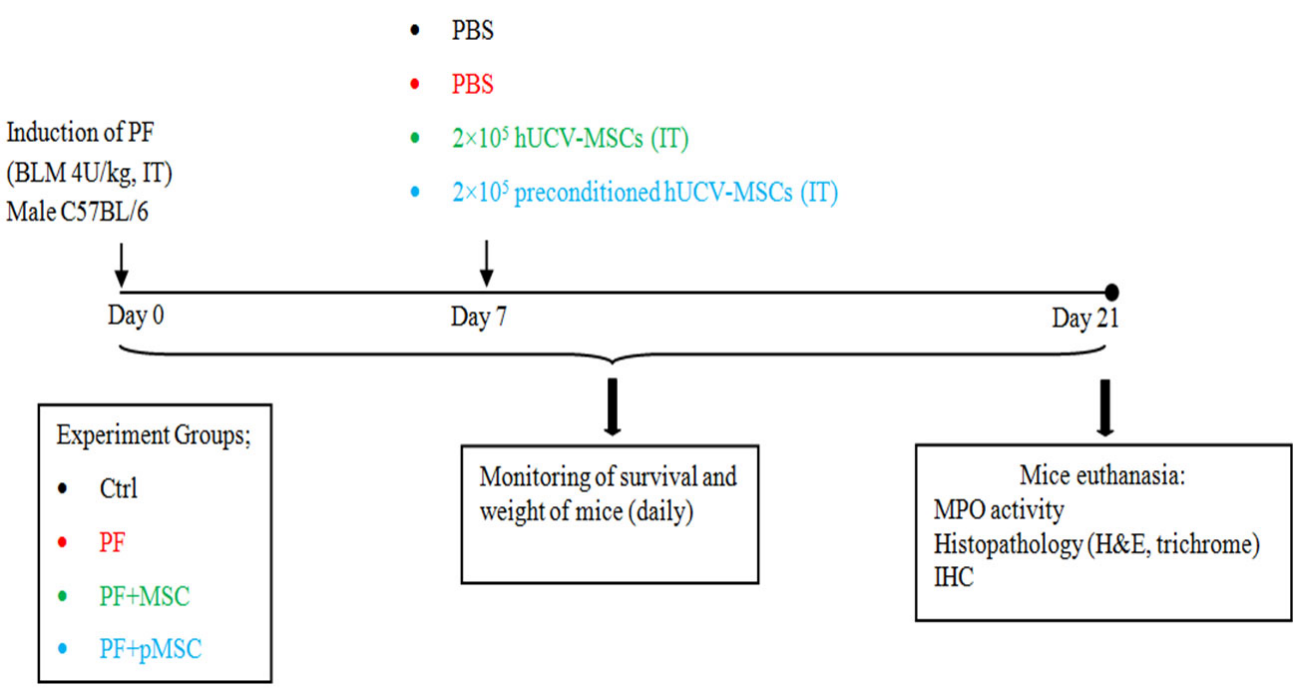
Study design for pulmonary fibrosis induction and treatment procedure. PF: pulmonary fibrosis, BLM: bleomycin, IT: intratracheally, Ctrl: control, MSC: mesenchymal stem cell, pMSC: preconditioned MSC, MPO: myeloperoxidase, H&E: hematoxylin-eosin, IHC: immunohistochemistry.

One week after bleomycin injection, the mice were euthanized, and hUCV-MSCs were prepared for administration to Mice. Both the hUCV-MSCs and H_2_O_2_ preconditioned hUCV-MSCs (p-MSC), at passage 4 with a concentration of 2×10^5^ cell/mouse, were suspended in 50 µL sterile PBS and administered intratracheally. The control groups received only PBS in the treatment time. The survival rate and weight of mice were recorded every day, and mice were sacrificed 14 days after the treatment. Lung tissues of the mice were removed promptly, shared in several parts, fixed in formalin for histopathological and immunohistochemical (IHC) examinations or frozen at -70°C in order to investigate MPO enzyme activity. Design of study is demonstrated in Figure 1.

### Histopathological examination

Formaldehyde-fixed (10% buffered formalde­hyde) lung tissues were embedded in par­affin (5 μm) and stained with hematoxylin-eosin (H&E) to investigate the volume of alveolar space and connective tissue. In addition, Masson’s trichrome was applied to evaluate collagen deposition and connective tissue mass. The lung tissue sections were examined using the light microscopy at ×100 and ×400 magnifications. Our histologist colleague (MJ.R) was blinded to each group and performed the histopathological evaluation of lung sec­tions. Moreover, in order to exactly investigate the histopathological changes, the volume of the alveolar space and connective tissue in all of the experimental groups were assessed applying morphometric analysis (graticule checkerboard 18×kpl-w12.5).^8,31^

### IHC examination

5-μm sections were obtained from paraffin-embedded lung samples and immunostained for TGF-β1 and α-SMA as fibrosis biomarkers. Briefly, samples were deparaffinized after the hydration, and antigen retrieval buffer was used for 40 minutes. Endogenous peroxidase was quenched with 0.1% hydrogen peroxide for 15-20 minutes. The samples were blocked using serum-free protein (Sigma Aldrich), then incubated with polyclonal antibodies of anti-mouse TGF-β1 (Santa Cruz Biotechnology (SC-146), 1:200 in PBS) and anti-mouse α-SMA (Abcam (ab-5694), 1:200 in PBS) overnight. Following washing with PBS, sections were detected with a sheep anti-rabbit IgG secondary antibody, followed by a peroxidase polymer and a solution of 0.1% 3, 3-diaminobenzidine (DAB) and 0.02% H_2_O_2_. Finally, all the slides were stained with hematoxylin to detect the nucleus of the cells.^32-34^

### Myeloperoxidase (MPO) enzyme activity

Neutrophil accumulation within the lung tissues was investigated by measuring the tissue MPO activity that was described previously.^6,35^ Frozen tissue samples were thawed at 4°C, then the tissues were homoge­nized in 20 mmol/L phosphate buffer (pH 7.4) and centrifuged (13 000 × g, 10 min, 4°C). The obtained pellet was resuspended with 0.5% hexadecyl trimethyl ammonium bromide (Sigma-Aldrich) in 50 mmol/L phosphate buffer (pH 6.0). Freeze-thaw cycles were done on the suspension four times, followed by sonication. The samples were recentrifuged (13 000 × g, 8 min, 4°C). MPO activity in the resulting supernatant was measured using TMB for ELISA at 405 nm. The results were reported as the absorbance per mg of lung tissue weight (A°/mg).

### Statistical analysis

Data was analyzed applying Graph Pad Prism 5 software. All data was represented as a mean ± standard deviation from the total number of mice in this study. Normality of all data has been checked with Kolmogorov-Smirnov normality test and differences between groups were analyzed by one-way ANOVA and Tukey post test. *P*< 0.05 was demonstrated statisti­cal significance.

## Result and Discussion

### Characterization of human UCV-MSCs by flow cytometry analysis and differentiation assay

**Figure 2:**
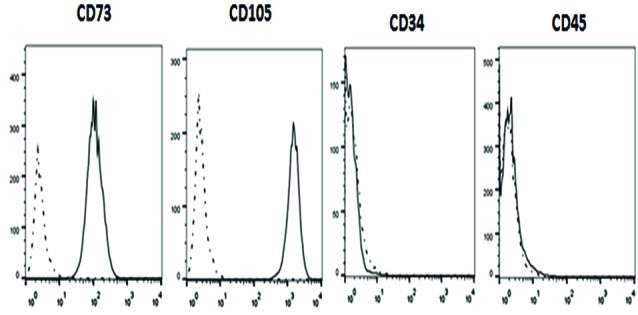
Characterization of human umbilical cord vein-derived mesenchymal stem cells (hUCV-MSCs) by flow cytometry analysis shows that these cells express high levels of CD73 and CD105 (mesenchymal markers) and low levels of CD34 and CD45 (hematopoietic markers).

**Figure 3:**
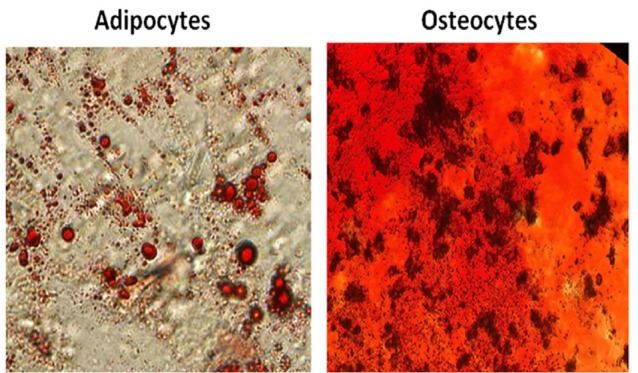
Characterization of human umbilical cord vein-derived mesenchymal stem cells (hUCV-MSCs) by differentiation assay shows that these cells can differentiate into adipocytes and osteocytes..

MSCs at passages two were analyzed with regard to the high expression level of the mesenchymal markers (CD73 and CD105) and lack of expression of the hematopoietic markers (CD34 and CD45) (Figure 2). In addition, differentiation potency of these cells is demonstrated by staining osteocytes and adipocytes with Alizarin Red S and Oil Red O, respectively (Figure 3).

### Survival and weight monitoring

The survival rate in all mice is similar between experimental groups (data not shown). All mice were sacrificed at the end of the experiment time (21 d) to perform histopathological and immunological investigations. In addition, the results of mice weight monitoring demonstrate no significant difference between PF and treated groups (PF+MSC, PF+pMSC) (data not shown).

### Histopathological findings

**Figure 4:**
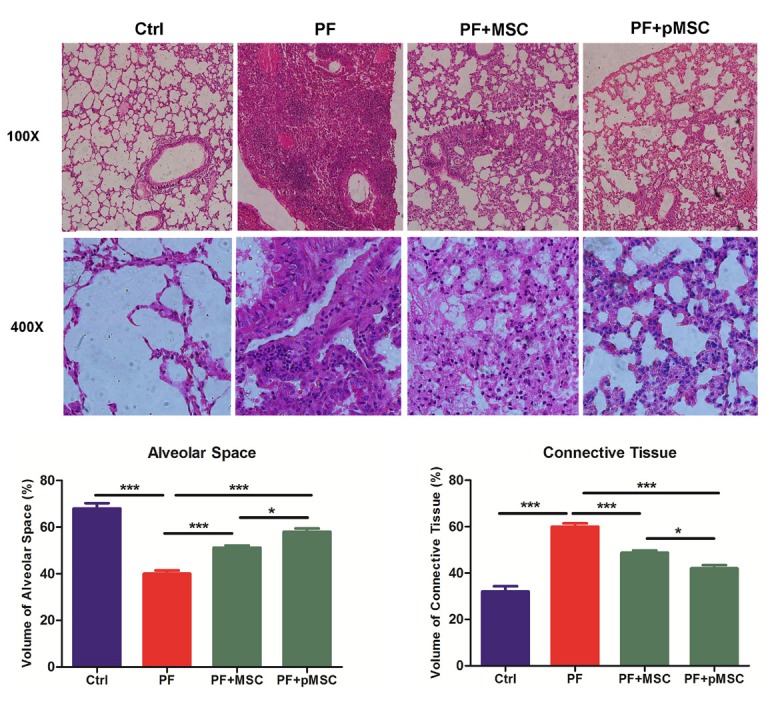
Microscopic analysis of lung sections after H&E staining. Compared to the pulmonary fibrosis group (PF), MSCs and preconditioned MSCs injection increased alveolar space and reduced connective tissue (*P* < 0.001). Also p-MSCs transplantation results in significantly decreased connective tissue (*P* < 0.05) and increased alveolar space (*P* < 0.05) in compared to the MSC group. H&E: hematoxylin-eosin, Ctrl: control, PF: pulmonary fibrosis, PF+MSC: treated group by hUCV-MSCs, PF+pMSC: treated group by preconditioned hUCV-MSCs, (Magnification 100× and 400×). Values are expressed as mean ± SE (n =7). (*** *P* < 0.001, ** *P* < 0.01, * *P* < 0.05).

The results of H & E staining at day 21 show a significant decrease of alveolar space and increase of connective tissue in mice receiving bleomycin (PF group), in comparison to the negative control group (Ctrl) (*P* < 0.001). In addition, infiltration of inflammatory cells, fibroblasts, and consequent diffuse fibrosis confirmed the induction of PF using BLM. In the group treated with hUCV-MSCs (PF+MSC) and preconditioned MSCs (PF+pMSC), the alveolar space is significantly increased (*P* < 0.001), while the connective tissue is significantly lower compared to the positive control group (PF) (*P* < 0.001). Also, compared to the PF+MSC group, preconditioned MSCs transplantation results in significantly decreased connective tissue (*P* < 0.05) and increased alveolar space (*P* < 0.05) (Figure 4).

**Figure 5:**
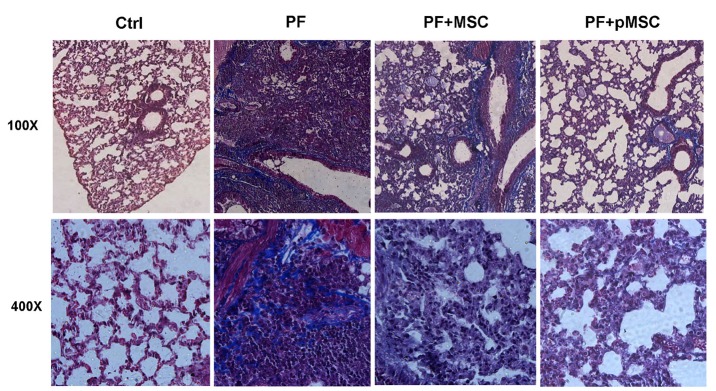
Microscopic analysis of lung sections after Masson’s trichrome staining. Compared to the pulmonary fibrosis group (PF), MSCs and especially preconditioned MSCs injection reduced collagen deposition (blue color) and extracellular matrix. Ctrl: control, PF: pulmonary fibrosis, PF+MSC: treated group by hUCV-MSCs, PF+pMSC: treated group by preconditioned hUCV-MSCs, (Magnification 100× and 400×).

In addition, Masson’s trichrome was applied to evaluate the ECM macromolecules, such as collagen deposition. The results of trichrome staining at day 21 show a considerable increase of the collagen deposition (blue color) in the PF group in compared to the Ctrl group. In the groups treated by hUCV-MSCs (PF+MSC), and especially preconditioned MSCs (PF+pMSC), a remarkable decrease of collagen deposition compared to the positive control group (PF) is observed (Figure 5).

### Immunohistochemical analysis of TGF-β1 and α-SMA expression

**Figure 6:**
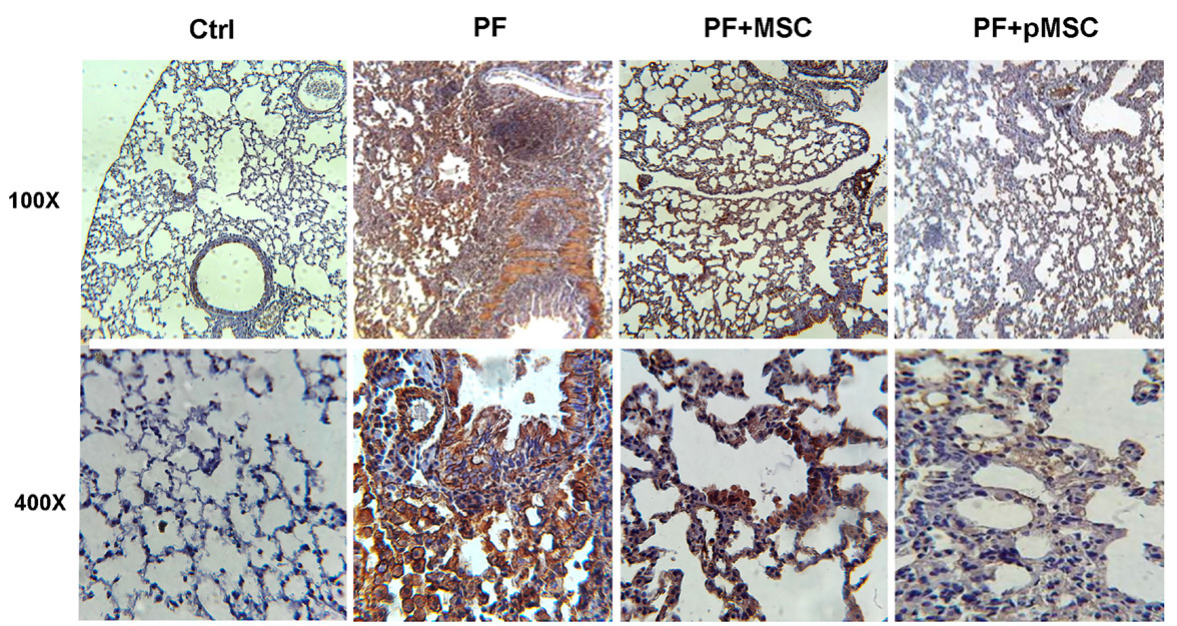
Representative images of TGF-β1 expression and localization in the lung sections after IHC staining. Compared to the pulmonary fibrosis group (PF), MSCs and especially preconditioned MSCs injection considerably decreased TGF-β1 protein expression in the parenchyma areas of lung tissues. IHC: Immunohistochemistry, Ctrl: control, PF: pulmonary fibrosis, PF+MSC: treated group by hUCV-MSCs, PF+pMSC: treated group by preconditioned hUCV-MSCs, (Magnification 100× and 400×).

**Figure 7:**
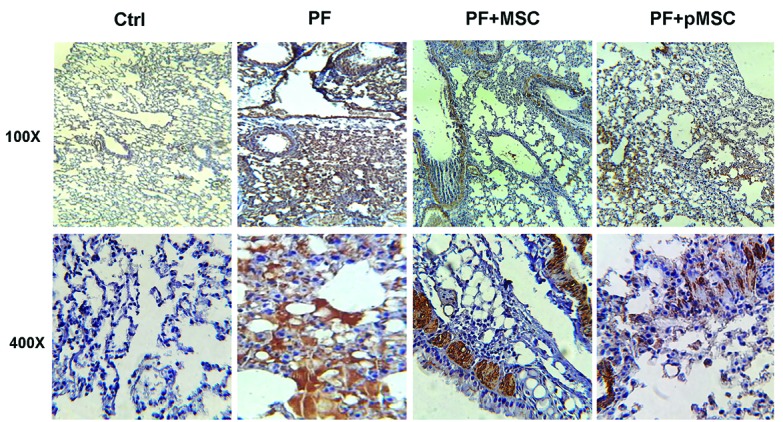
Representative images of α-SMA expression and localization in the lung sections after IHC staining. Alpha-SMA expression was mainly localized in the pulmonary vessel walls in the negative control (Ctrl) group, while in the pulmonary fibrosis (PF) group was localized in the parenchyma of fibrotic areas. Compared to the pulmonary fibrosis group (PF), MSCs and especially preconditioned MSCs injection considerably decreased α-SMA protein expression in the parenchyma areas of lung tissues. IHC: Immunohistochemistry, Ctrl: control, PF: pulmonary fibrosis, PF+MSC: treated group by hUCV-MSCs, PF+pMSC: treated group by preconditioned hUCV-MSCs, (Magnification 100× and 400×).

According to IHC staining results, there is a high expression of the TGF-β1 and a-SMA protein in the parenchyma areas of lung tissues from PF group compared to the negative control (Ctrl) group. However, the group administered with hUCV-MSCs (PF+MSC) and preconditioned MSCs (PF+pMSC) show a remarkable decrease of the TGF-β1 and a-SMA protein expression compared to the positive control group. In addition, α-SMA expression is mainly localized in the pulmonary vessel walls in the negative control (Ctrl) group, whereas in the PF group was localized in the parenchyma of fibrotic areas (Figures 6 and 7).

### MPO enzyme activity assay

**Figure 8:**
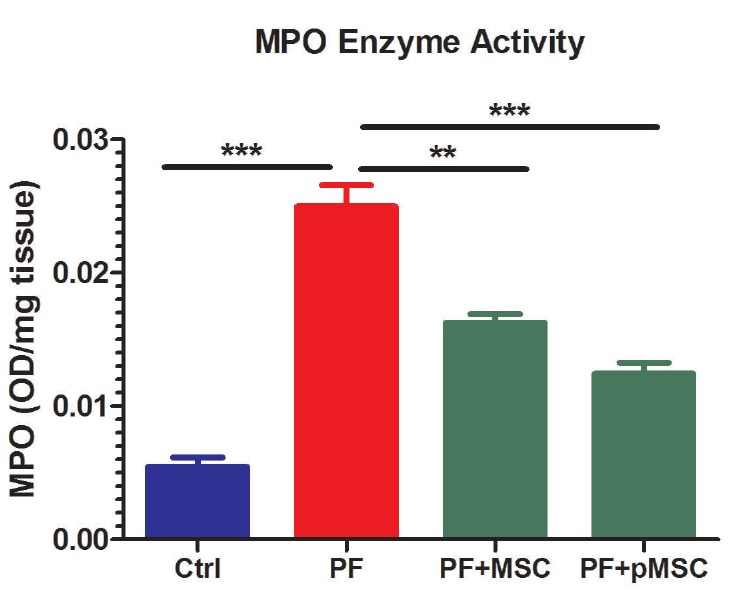
Effect of hUCV-MSCs and preconditioned hUCV-MSCs on myeloperoxidase (MPO) activity in lung tissue. The results were reported as the absorbance per mg of lung tissue weight (OD/mg). Values are expressed as mean ± SE (n = 7). MPO activities following BLM injection (PF group) were significantly higher than the control (Ctrl) group (*P* < 0.001). Therefore, hUCV-MSCs (*P* < 0.01) and preconditioned MSCs (*P* < 0.001) demonstrated a significant decrease of MPO activities in the treated (PF+MSC and PF+pMSC) groups as compared to the PF group. However, there was no significant difference between the two treated groups. (*** *P* < 0.001, ** *P* < 0.01)

MPO has been known as a valuable biomarker of lung inflammation and injury. MPO enzyme activity confirms the oxidative stress following BLM exposure and extent/activation of inflammatory cells, such as neutrophils or monocyte/macrophages in lung tissue.^6^ Accordingly, Figure 8 demonstrates a five-fold increase in MPO activities following BLM injection (PF group) as compared to the negative control (Ctrl) group (*P* < 0.001). HUCV-MSCs and preconditioned MSCs significantly decrease MPO activities in the treated groups (PF+MSC (*P* < 0.01) and PF+pMSC (*P* < 0.001)) as compared to the PF group. Yet, no significant difference is observed between the two treated groups.

PF is a progressive inflammatory and fibrotic lung disorder, distinguished by overexpression of pro-inflammatory cytokines, reactive oxygen species and pro-fibrotic cytokines, such as TGF-β1. Deposition of ECM proteins, like collagen, aggravates the lung injury. Moreover, the connective tissue will reduce the alveolar space of the lung.^1,8,31^ To date, there is no useful cure for PF. The only possible treatment approach for PF patients at the end stage of the disease is lung transplantation. However, PF is a life-threatening disease.^36^ Regarding fibrosis immunopathogenesis, many previous studies have revealed that MSCs, because of anti-inflammatory and anti-fibrotic effects, could be potentially helpful for the treatment of PF.^8,36-39^ Nevertheless, the inappropriate environment of inflammatory and fibrotic tissue with large amounts of pro-inflammatory cytokines, reactive oxygen species, and many other harsh factors will diminish the potency of cell therapy after transplantation.^40-42^ Some studies reported that *in vitro* preconditioning of MSCs could promote essential features, such as survival, migration and tissue repair capacity of transplanted stem cells against harsh microenvironment.^14,25, 27-29^ Regarding the previous studies and the necessity of conducting *in vivo* study,^27,29^ our work is among the few studies ever done in this field on experimental animal models.^14,30^

In the current study, we are the first to evaluate the anti-inflammatory and anti-fibrotic effects of H_2_O_2_ preconditioned hUCV-MSCs in mice with PF induced by bleomycin. H_2_O_2_ preconditioned cells show increased survival, proliferation and grafting rate. Upregulation of several anti-apoptotic proteins, growth factors, cytokines and activation of some signaling pathways is involved *in vitro* H_2_O_2_ effects, whereas there are few pieces of information *in vivo* investigations in this field.^27,29^ Results of this work suggest that intratracheal transplantation of H_2_O_2_ preconditioned hUCV-MSCs are more effective in decreasing connective tissue and collagen deposition and increasing alveolar space in the experimental PF model (Figures 4 and 5). In addition, H_2_O_2_ treated MSCs are more effective in reducing TGF-β1 and α-SMA expression in fibrotic lung tissue (Figures 6 and 7). However, there was no significant difference between the H_2_O_2_-treated MSCs and PF+MSC group in MPO enzyme activity (Figure 8).

Several studies examined the effects of the route of MSC administration in animal PF. These studies showed that local (IT) and systemic (IV or IP) injection of MSCs attenuate lung injury and fibrosis.^14,38^ Nevertheless, local injection requires a lesser amount of these cells and reduces systematic administration complications.^38,43,44^ Therefore, in this study, 2×10^5^ H_2_O_2_ preconditioned hUCV-MSCs (PF+pMSC) and the same dose of untreated hUCV-MSCs (PF+MSC) was injected intratracheally (IT) into the C57BL/6 mice with bleomycin-induced PF. In many previous studies in this field, MSC transplantation was conducted within the first 2 weeks after BLM injection. In the Moradi et al^8^ study, administration of MSCs was done 15 min after BLM injection, whereas in this work administration of these cells performed on the seventh day after PF induction. Similar to the results of previous studies, our results show that MSCT in the initial or inflammatory phase can have useful effects, rather than the fibrotic phase of PF.^14,38,45^ There are a few studies to address the involved mechanisms of H_2_O_2_ preconditioning. Nouri et al^29^ noted that preconditioning of MSCs with 20 μM H_2_O_2_ (12 h) improved cell resistance against lethal microenvironment via HIF-1α overexpression. Also, Bashiri et al^27^ demonstrated that preconditioning with 15 μM (24 hours) has better protective effects than other concentrations of H_2_O_2_. For this reason, we used 15 μM (24 hours) for preconditioning of the hUCV-MSCs in the present study.

## Conclusion

The results of this study reveal that *in vitro* preconditioning of MSCs with a sub-lethal concentration of H_2_O_2_ can improve the therapeutic potency of MSC therapy in IPF and diminish inflammatory and fibrotic factors in bleomycin-induced PF. Nevertheless, further examinations should be conducted to evaluate the anti-inflammatory and anti-fibrotic effects of H_2_O_2_ preconditioned MSCs in other experimental models of diseases. In addition, future studies are required to investigate the cytoprotective mechanisms involved during the *in vitro* preconditioning.

## Conflict of Interest

The authors declare that they have no conflict of interest.

## Ethical Issues

All of the animal studies were performed under approval of the Ethics Committee of the Kurdistan University of Medical Sciences, Sanandaj, Iran (Project number: 1392.106, Approval date: Feb 16, 2014).

## Acknowledgments

This study was supported by the Cellular and Molecular Research Center at the Kurdistan University of Medical Sciences [grant number 1392.106]. We are grateful to the Maryam Moradi in the Immunology Department of the Kurdistan University of Medical Sciences, for the technical assistance in this project.
